# Preferential Mechanochemical Activation of Short Chains in Bidisperse Triblock Elastomers

**DOI:** 10.1021/acsmacrolett.3c00366

**Published:** 2023-08-24

**Authors:** Zijian Huo, Kasey F. Watkins, Brandon C. Jeong, Antonia Statt, Jennifer E. Laaser

**Affiliations:** † Department of Chemistry, University of Pittsburgh, Pittsburgh, Pennsylvania 15260, United States; ‡ Department of Chemical and Biomolecular Engineering, University of Illinois, Urbana−Champaign, Illinois 61801, United States; § Department of Materials Science and Engineering, University of Illinois, Urbana−Champaign, Illinois 61801, United States

## Abstract

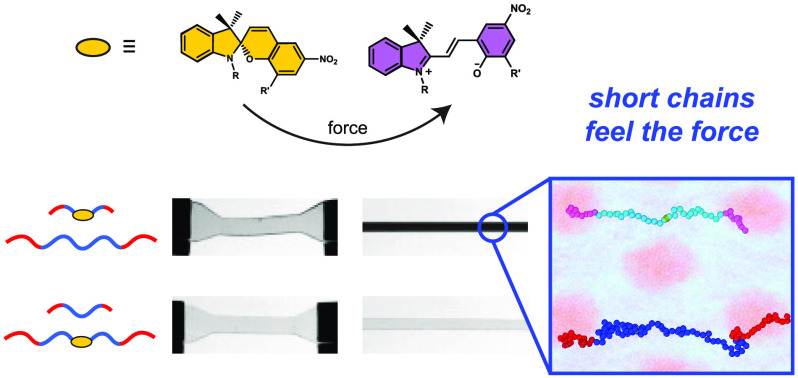

Polymer mechanochemistry offers attractive opportunities for using macroscopic forces to drive molecular-scale chemical transformations, but achieving efficient activation in bulk polymeric materials has remained challenging. Understanding how the structure and topology of polymer networks impact molecular-scale force distributions is critical for addressing this problem. Here we show that in block copolymer elastomers the molecular-scale force distributions and mechanochemical activation yields are strongly impacted by the molecular weight distribution of the polymers. We prepare bidisperse triblock copolymer elastomers with spiropyran mechanophores placed in either the short chains, the long chains, or both and show that the overall mechanochemical activation of the materials is dominated by the short chains. Molecular dynamics simulations reveal that this preferential activation occurs because pinning of the ends of the elastically effective midblocks to the glassy/rubbery interface forces early extension of the short chains. These results suggest that microphase segregation and network strand dispersity play a critical role in determining molecular-scale force distributions and suggest that selective placement of mechanophores in microphase-segregated polymers is a promising design strategy for efficient mechanochemical activation in bulk materials.

Polymer mechanochemistry has emerged as a promising approach for stimulus-responsive control of chemical reactions.^1−4^ When force is applied across a chemical bond, the potential energy landscape of that bond shifts, facilitating bond breakage and formation of products that would otherwise be kinetically or thermodynamically disfavored.^5^ In the past decade, the development of new force-responsive chemistries has enabled new classes of useful stimulus-responsive materials,^4^ including materials that provide a visual readout of stress and/or strain,^6−9^ materials that self-heal after mechanical damage,^10−13^ and materials that offer precise, targeted release of small-molecule payloads,^14−16^ to name only a few.

Polymer networks should be a useful platform for driving mechanochemical activation on large numbers of molecules at once. However, yields of force-driven reactions in elastomeric polymer networks are typically low, even for mechanophores that exhibit high activation yields in solution sonication experiments.^17,18^ Structural and topological heterogeneity have recently been identified as key factors that may reduce the efficiency of force transmission from the macroscopic to the molecular scale.^19^ In particular, spatial heterogeneity in the distribution of cross-links, defects, and dispersity in the network strand lengths may all contribute to broad molecular-scale force distributions that lead to activation of mechanophores in only the small number of strands experiencing the highest force.^20^ Understanding how network structures and chain conformations influence molecular-scale force distributions and how polymer architectures can be designed to direct the force to specific network strands is critical for designing materials with optimized activation yields and precisely tailored activation profiles.

Here, we use a combination of experiments and computer simulations to investigate how network strand dispersity impacts molecular-scale force distributions and mechanochemical activation in self-assembled triblock copolymers. As illustrated in Figure 1, we exploit the self-assembly of bidisperse triblock copolymer blends to generate network-like structures with mixtures of short and long chains. In these materials, self-assembly of the glassy end-blocks creates physical cross-links between the chains, which transmit force to the rubbery midblock and drive activation of mechanophores in the centers of the polymer chains.^21−23^ By changing which component of the blend contains mechanophores (short chains, long chains, or both), we are effectively able to “label” and probe the activation of chains with different elastic strand lengths within otherwise equivalent networks. We find that activation occurs primarily in the short chains, which molecular dynamics (MD) simulations reveal is driven in large part by pinning of the elastically effective midblock ends to the glassy/rubbery interface. These results suggest that controlling both microphase segregation and network strand length distributions is an important design strategy for directing molecular-scale force distributions and enabling efficient mechanochemical activation in bulk materials.

**Figure 1:**
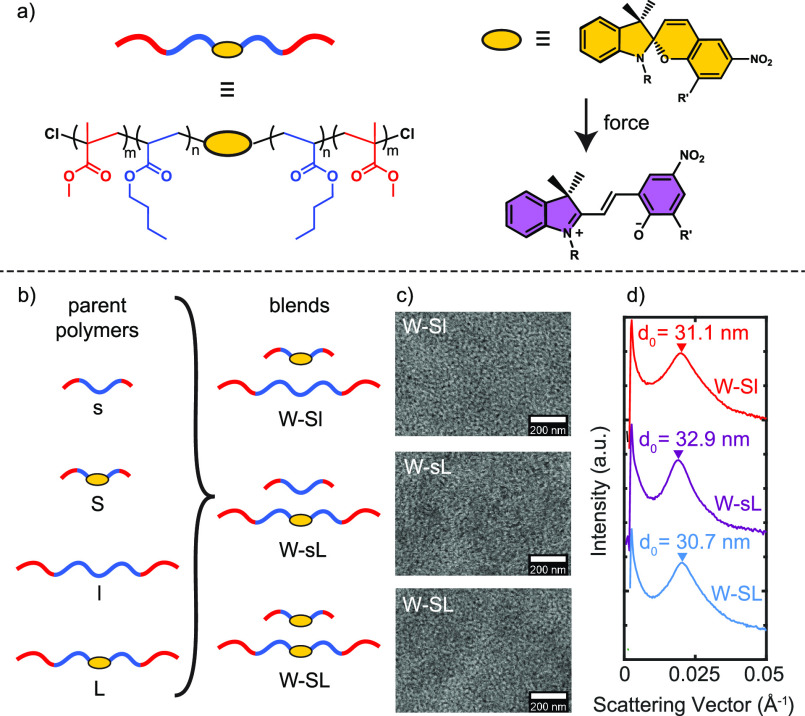
(a) Chemical structures of spiropyran-containing triblock copolymers and the mechanochemical isomerization of spiropyran (colorless) into merocyanine (blue/purple), (b) summary of parent polymers and bidisperse blends, and (c) STEM images and (d) SAXS patterns of blends prepared by mixing equal masses of short and long polymers with and without mechanophores. Full characterization data for the parent polymers and blends are provided in the Supporting Information.

We first synthesized four triblock copolymers with glassy poly(methyl methacrylate) (PMMA) end blocks and rubbery poly(*n*-butyl acrylate) (PnBA) midblocks, as depicted in Figure 1. All polymers had approximately the same volume fraction of PMMA (*f*_PMMA_ ≈ 0.45) and narrow dispersities (*Đ* ≈ 1.2), but differed in overall molecular weight (*M*_n_ ≈ 60 or 150 kg/mol) and whether they contained a spiropyran mechanophore in the center of the PnBA midblock. These parent polymers were then blended to prepare bidisperse samples with equal weight fractions of short and long chains, but with spiropyran mechanophores in different subpopulations of the chains. Samples labeled W-Sl contained mechanophores only in the short (S) chains; samples labeled W-sL contained mechanophores only in the long (L) chains; and samples labeled W-SL contained mechanophores in both the short and the long (SL) chains. Here, the “W-” prefix is used to indicate that the samples contained equal weight fractions of short and long chains; complementary experiments on samples with equal number fractions of short and long chains (“N-”) are reported in the Supporting Information. Size exclusion chromatography confirmed that all three blended samples had similar number-average molecular weights and molecular weight distributions. Scanning transmission electron microscopy (STEM) images and small-angle X-ray scattering (SAXS) patterns of the self-assembled samples revealed that all three polymer blends self-assembled into disordered, microphase-segregated materials with domain spacings on the order of 30 nm, consistent with the disordered, bicontinuous morphologies observed in other polydisperse block copolymer systems.^24−26^

The mechanical properties and mechanochemical activation profiles of the materials were then characterized by measuring changes in their optical absorption between 550 and 630 nm during uniaxial tensile deformation.^23^ The stress–strain and activation–strain profiles of all three blends are shown in Figure 2. As seen in Figure 2a, all three blends exhibited elastic behavior at low strain, followed by plastic deformation and finally strain hardening. The presence of a distinct yield point, followed by plastic deformation, suggests that the mechanical response at low strain is dominated by deformation of the glassy PMMA phase. While the moduli of the samples varied by up to 30%, likely due to slight variations in the molecular weight distributions, the shapes of the stress–strain curves were comparable for all three samples. Their activation profiles, shown in Figures 2b–d, however, differed substantially. While activation occurred only after the yield point in all samples, as has been observed in glassy networks,^27^ the W-Sl samples and W-SL samples (both of which contained mechanophores in the short strands) activated relatively early, with the onset of activation observed around ϵ ≈ 0.67 and σ ≈ 6 MPa. The W-sL samples, on the other hand, which only contained mechanophores in the long strands, activated much later, with the onset of activation not observed until ϵ ≈ 2.25 and σ ≈ 9 MPa. The total activation of the W-SL sample, which contained mechanophores in both the short and the long chains, was also almost identical with that of the W-Sl sample, which contained mechanophores in only the short chains. Similar trends were observed in samples containing equal number fractions of short and long chains (see Supporting Information). Together, these results reveal that in block copolymer elastomers mechanochemical activation is almost entirely dominated by the short chains (even when both populations of chains contain mechanophores), suggesting that the molecular-scale forces on these chains are much higher than those on their longer counterparts.

**Figure 2:**
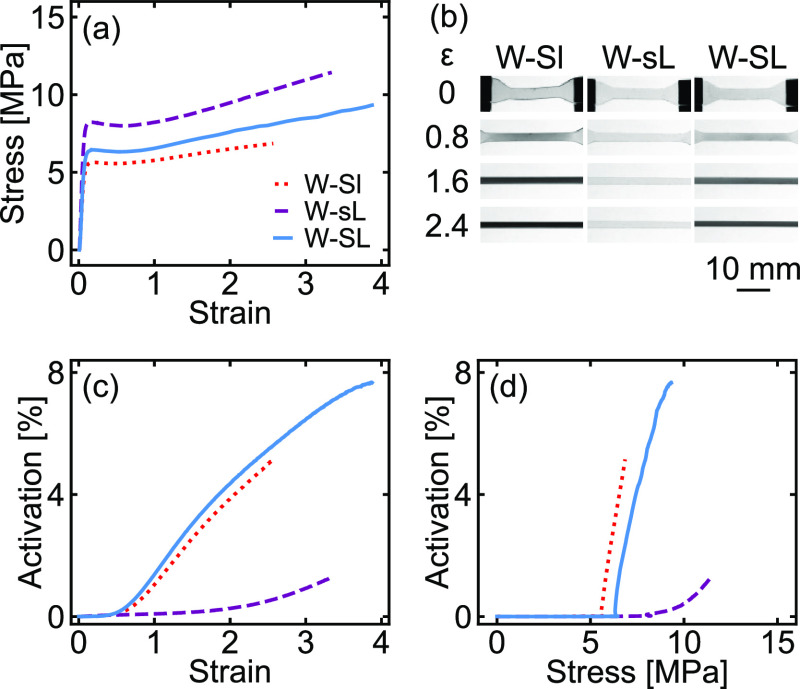
(a) Stress–strain profiles of bidisperse polymer blends, (b) snapshots from videos obtained during tensile deformation illustrating the absorption changes in the 550–630 nm range arising from mechanophore activation, and the corresponding (c) activation–strain and (d) activation–stress profiles. Percent activation is normalized to the total number of chains in each sample. Stress and strain values for all experimental data are engineering stress and engineering strain, unless otherwise noted.

To understand the molecular-scale origins of this result, we carried out coarse-grained molecular dynamics simulations on triblock copolymers with bimodal molecular weight distributions designed to mimic the experimental system.^28^ The PMMA and PnBA block fractions were chosen to yield well-defined cylindrical morphologies, as shown in Figure 3, which capture most of the local structural features expected in the experimental systems. These equilibrated morphologies were then subjected to uniaxial deformation,^29,30^ and activation of the mechanophores in both the long and the short chains was quantified by monitoring changes in the bond length of a double-well unit in the center of the chains. The resulting activation profiles for each subset of chains are shown in Figure 3. As seen in this figure, the simulations yielded overall activation profiles similar to those observed in the experiments. Throughout the deformation, activation of the short chains was higher than the activation of the long chains when compared at either the same strain (Figure 3c) or at the same stress (Figure 3d), and the short chains exhibited an earlier onset of activation (ϵ_true_ ≈ 0.75) than the long chains did (ϵ_true_ ≈ 0.95). The overall activation also closely tracked that of the short chains alone until relatively high strains (ϵ_true_ > 1). While the simulations were run on perfectly ordered, anisotropic morphologies and thus likely overestimate the overall response of more disordered and/or isotropic samples, the close qualitative agreement of the simulation data with the experimental results validates the simulation model and reinforces the conclusion that mechanochemical activation is much more efficient in the shorter chains.

**Figure 3:**
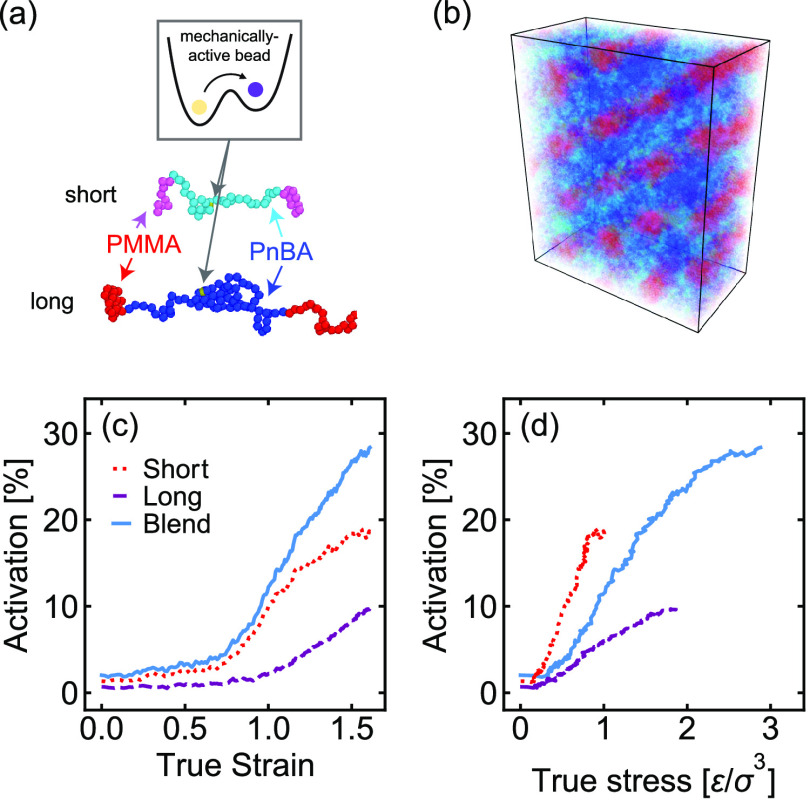
(a) Setup of the Kremer–Grest bead–spring model used in the coarse-grained simulations, including the double-well potential used to represent the mechanophore, (b) snapshot of a representative cylindrical morphology obtained for the bidisperse polymer blends, and the resulting (c) activation–strain and (d) activation–stress profiles. Percent activation is normalized to the total number of chains in each sample.

Detailed analysis of the MD simulations provides insights into the molecular-scale features that promote this preferential activation of the short chains. As shown in Figure 4a, activation occurs primarily in the tie chains connecting different glassy domains, with the short tie chains exhibiting a higher overall activation than the long tie chains. Analysis of the tie chain conformations reveals that the midblocks of the short tie chains extend to a fraction of their contour lengths that is larger than that of the midblocks of the long tie chains (Figure 4b), with activation occurring primarily in the most highly stretched portion of the tie chain population. This higher fractional extension corresponds to a higher force on these PnBA midblocks. As illustrated in Figure 4c, this difference in chain extension occurs because the ends of the rubbery midblocks are “pinned” to the glassy/rubbery interface and the short tie chains must stretch more than the long chains to span the same distance between adjacent interfaces. Analysis of the chain orientations (see Supporting Information) additionally reveals that activation is higher in chains that are well-aligned with the pulling direction and that the short tie chains align more easily than the long tie chains do. As in the experimental systems, similar trends were also observed in samples containing equal number fractions of short and long chains.

**Figure 4:**
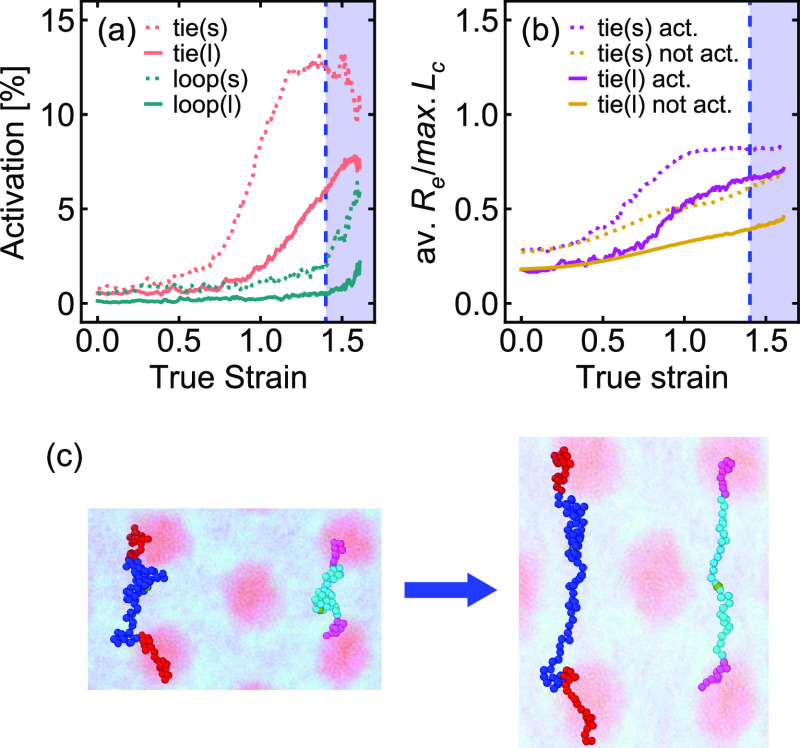
(a) Activation–strain data from MD simulations broken down by chain length and chain type, (b) average fractional extension of the midblocks of each chain type during deformation, and (c) simulation snapshot showing a single short chain and a single long chain in the undeformed (left) and deformed (right) states. The shaded areas indicate strains at which cavitation was observed.

Both the experimental and computational results reported in this work show that mechanochemical activation preferentially occurs in the short tie chains in bidisperse triblock elastomers. Preferential activation of the short tie chains is so pronounced that the short chains dominate the overall activation profile of the material, even though the short chains have a lower probability of forming tie chains than the long chains do (see Supporting Information). From a fundamental perspective, since the mechanophores act as molecular-scale force probes, these results provide detailed new insights into how force is distributed in block copolymer elastomers. In particular, short tie chains carry much of the load, even when they constitute a minority of the tie chains in the material. Interestingly, this result is in contrast to the behavior observed in linear homopolymers, where activation is typically more pronounced in longer chains,^31,32^ and in both rubbery and glassy cross-linked networks, where it has been shown that activation levels are essentially independent of the cross-linker length as long as the probe strands are shorter than the average strand length of the material.^27,33^ This suggests that pinning of the midblock ends to the hard/soft interface may be critical for directing force to the shorter strands. While similar effects might be expected to occur in covalently cross-linked rubbers, relaxation of the junction points in these materials may also reduce the preferential stretching of the short chains,^33^ and further investigation will be needed to understand how network topology and strand dispersity affect mechanochemical activation in those materials. We note, however, that the absolute activation in the block copolymer samples appears to be higher than in most elastomers reported to date.^17,18^ While there is a small amount of uncertainty in the absolute activation arising from the measurement of the extinction coefficient of the activated mechanophores (see Supporting Information), this result suggests that pinning of the midblocks to the glassy/rubbery interface may facilitate a more efficient force transfer overall. The observation that activation only occurs after the yield point of the materials further highlights the importance of the glassy phase, with rearrangement of the glassy phase (which is not captured in the simulations) necessary prior to stretching and mechanophore activation in the rubbery midblock strands.^27^

From an applied perspective, this work also suggests that microphase segregation, control of elastic strand dispersity, and carefully considered placement of mechanophores in different populations of the network strands can be used to optimize both the overall mechanochemical activation efficiencies in polymeric materials and the point at which activation occurs during a macroscopic deformation. To facilitate early activation, mechanophores should be placed in the short strands of a triblock elastomer, while materials displaying delayed activation can be prepared by instead placing mechanophores in the long strands. Additionally, because the mechanophores in the long strands display a much lower overall activation and are in some sense “wasted” mechanophores, the overall activation yield per mechanophore can be improved by placing the mechanophores primarily in the short strands. Regardless of the targeted activation profile, however, the formation of microphase-segregated morphologies and pinning of the elastic chain ends to the block interface appear to be critical for forcing early extension of the short midblocks, and we anticipate that controlling microphase segregation should be a powerful design parameter for enhancing mechanochemical activation in elastomeric networks.

In summary, we showed that in microphase-segregated triblock elastomers with bidisperse molecular weight distributions, mechanochemical activation occurs primarily in the short chains. These results suggest that network strand dispersity plays an important role in determining molecular-scale force distributions in these materials and that selective placement of mechanophores in the highest-force or highest-extension strands can be used to both control the onset of activation and improve overall activation yields. Further investigation of how microphase segregation, network topology, dispersity, and defect content influence molecular-scale force distributions and mechanochemical activation yields promises to be a fruitful avenue for achieving efficient mechanochemical activation in bulk polymer materials.

## Supporting Information Available

Detailed experimental and computational methods, characterization data for all synthesized polymers and polymer blends (NMR, SEC, STEM, SAXS, and additional tensile tests), and supplemental analysis of MD simulations (PDF)
