# Supplementary material for: Preferential Mechanochemical Activation of Short Chains in Bidisperse Triblock Elastomers
Source: ACS Macro Lett. 2023 Aug 24;12(9):1213–7. doi: 10.1021/acsmacrolett.3c00366 (PMC10515626; doi:10.1021/acsmacrolett.3c00366)
Supplement: Supplementary file 1 — mz3c00366_si_001.pdf [file mz3c00366_si_001.pdf]

Supporting Information: Preferential Mechanochemical Activation of Short Chains in Bidisperse Triblock Elastomers

Zijian Huo,† Kasey F. Watkins,† Brandon C. Jeong,‡ Antonia Statt,*,¶,‡ and Jennifer E. Laaser*,†

†*Department of Chemistry, University of Pittsburgh, Pittsburgh, PA 15260, USA*

‡*Department of Chemical & Biomolecular Engineering, University of Illinois, Urbana-Champaign, IL 61801, USA*

¶*Department of Materials Science & Engineering, University of Illinois, Urbana-Champaign, IL 61801, USA*

E-mail: statt@illinois.edu; j.laaser@pitt.edu

Contents

1 Methods **S-2**

1.1 Experimental Details S-2

1.1.1 Materials S-2

1.1.2 Polymer Synthesis S-3

1.1.3 Preparation of Polymer Blends S-5

1.1.4 Characterization S-5

1.2 Computational Details S-7
1.2.1 Molecular dynamics model S-8
1.2.2 Sample Generation S-8
1.2.3 Deformation S-9

2 Results **S-10**
2.1 Experimental Results S-10
2.1.1 Parent Polymers S-10
2.1.2 Equal Weight-Fraction Blends S-17
2.1.3 Equal Number-Fraction Blends S-21
2.2 Computational Results S-24
2.2.1 Equilibrated Morphologies S-24
2.2.2 Tensile and Activation Responses S-25
2.3 Comparison of Experimental and Computational Results S-29

References **S-31**

1 Methods

1.1 Experimental Details

1.1.1 Materials

2-bromopropionic acid, acetone, acetonitrile, ethyl ether anhydrous, hexanes, hydrochloric acid (37%), anhydrous magnesium sulfate, methanol, methylene chloride (DCM), potassium hydroxide, sodium bicarbonate, sodium chloride, sodium hydroxide, tetrahydrofuran (THF), toluene, and N, N′-dicyclohexylcarbodiimide (≥99%) (DCC), were purchased from Fisher Scientific. Ethanol was purchased from Decon Labs. 1,4-dioxane, 2-bromoethanol (95%), 2,6-dibromoheptanedioate (DMDBHD), 4-(dimethylamino)pyridine (≥99%) (DMAP), benzyl alcohol, *n*-butyl acrylate (nBA), copper(I) bromide (99.999%), copper(I) chloride (>99.995%),

ethyl acetate (99.5%), methyl methacrylate (MMA), and N,N,N′,N″,N″-pentamethyldiethylenetriamine (PMDETA) were purchased from Sigma-Aldrich. 2, 3, 3-trimethylindolenine (>97.0+%) and 3-chloromethyl-5-nitrosalicylaldehyde were purchased from TCI chemicals. Phosphotungstic acid was purchased from Electron Microscopy Sciences. Milli-Q water (18.2 MΩ·cm) was obtained from a Synergy water purification system (Millipore Sigma). The monomers (nBA and MMA) were filtered through activated neutral alumina immediately before use to remove the monomethyl ether hydroquinone inhibitors. The initiator, $SPBr_2$, was synthesized as previously reported.[S1–S3] All other reagents were used as received unless otherwise noted.

1.1.2 Polymer Synthesis

Synthesis of poly(*n*-butyl acrylate) macroinitiator (PnBA) and SP-functionalized PnBA macroinitiator (PnBA-SP) The PnBA and PnBA-SP macroinitiators were synthesized via ATRP from the DMDBHD and $SPBr_2$ difunctional initiators, respectively.[S3] In a typical polymerization using DMDBHD, copper(I) bromide (332 mg, 2.30 mmol, 2.0 eq.), nBA (118 g, 920 mmol, 800 eq.), DMDBHD (250 μL, 1.15 mmol, 1.0 eq.), PMDETA (480 μL, 2.30 mmol, 2.0 eq.), and anisole (30 mL) were combined in a Schlenk flask and degassed via three freeze-pump-thaw cycles. The flask was then filled with argon, and the reaction mixture was heated at 90 °C for 4 hours until the targeted conversion (monitored by ^{1}H-NMR) was reached. The resulting viscous solution was precipitated into a cold 50 % ethanol-water mixture, after which the polymer was re-dissolved in dichloromethane and passed through a silica column two times to yield a light yellow color viscous liquid. The polymer was then further dried in a vacuum oven at 50 °C to remove residual solvent. PnBA-SP macroinitiators were prepared in a similar manner using $SPBr_2$ as an initiator. Synthetic conditions for each macroinitiator are listed in Table S1, and characterization data is reported in Section 2.1.1, below. We note that a slight excess of the copper(I) bromide was used when preparing the mechanochemically active PnBA-SP polymers because the spiropyran mechanophore forms

a complex with the copper(I) ion and slows down the polymerization kinetics, necessitating a slight excess of copper to compensate.

Table S1: Preparation of PnBA and PnBA-SP macroinitiator

**Polymer**	Initiator Used	nBA[a]	Initiator[a]	PMDETA[a]	Cu(I)Br[a]	Time, h	Conversion, %
PnBA-s	DMDBHD	800[b]	1	2	2	4	30
PnBA-S	$SPBr_2$	800	1	2	2.3	3.5	32
PnBA-l	DMDBHD	800	1	2	2	6	61
PnBA-L	$SPBr_2$	800	1	2	2.4	5	63

[a] These values are in mole equivalent.
[b] The monomer concentration is [nBA] = 5.8 M.

Synthesis of PMMA-*b*-PnBA-*b*-PMMA (MBM) triblock copolymers Mechanically inactive (MBM) and mechanically active (MBM-SP) triblock copolymers were synthesized by chain extension of PnBA and PnBA-SP.[S3] In a typical synthesis, copper(I) chloride (81.1 mg, 0.819 mmol, 2.0 eq.), MMA (28.6 g, 286 mmol, 700 eq.), PnBA (12.0 g, 0.460 mmol, M_n = 26 kg/mol, 1.1 eq.), PMDETA (170 μL, 0.830 mmol, 2.0 eq.), and anisole (20 mL) were combined in a Schlenk flask and degassed via three freeze-pump-thaw cycles. The flask was filled with argon, and the reaction mixture was heated at 70 °C until the targeted composition was reached, as monitored by ^{1}H-NMR. The resulting crude product was precipitated into cold ethanol, re-dissolved in dichloromethane, and passed through a silica column two times before being dried under vacuum to yield a white polymer solid. MBM-SP triblock copolymers were prepared following a similar procedure, with PnBA-SP as the macroinitiator. Synthetic details for all triblock copolymers used in this study are provided in Table S2, and characterization data is reported in Section 2.1.1, below.

Table S2: Preparation of MBM and MBM-SP triblock copolymers

**Polymer**	Initiator Used	MMA[a]	Initiator[a]	PMDETA[a]	Cu(I)Cl[a]	Time, h	Conversion, %
MBM-s	PnBA-s	700[b]	1.1	2	2	2	47
MBM-S	PnBA-S	700[c]	1.1	2	2	2.5	36
MBM-l	PnBA-l	2000[d]	1.1	2	2	4.5	31
MBM-L	PnBA-L	2000[d]	1.1	2	2	5	32

[a] These values are in mole equivalents.
[b] [MMA] = 4.6 M.
[c] [MMA] = 4.3 M.
[d] [MMA] = 3.2 M.

1.1.3 Preparation of Polymer Blends

Bidisperse polymer blends were prepared with both equal number fractions (50:50 mol %) and equal weight fractions (50:50 wt. %) of the short and long polymers. Stock solutions of each parent polymer (ca. 17 wt.%) were prepared by dissolving the synthesized polymers in toluene. Appropriate volumes of each solution were mixed to obtain matched mole or mass ratios of the component polymers. After mixing, ∼8 mL of each polymer blend solution was deposited into a custom-built PTFE mold and dried overnight by slow evaporation at room temperature to generate a polymer film (ca. 0.5 ± 0.1 mm thick). The polymer film was then annealed under dioxane vapor at 50 °C for 48 hours and dried under vacuum overnight before further characterization.

1.1.4 Characterization

Proton nuclear magnetic resonance (^{1}H NMR) spectra were recorded on a Bruker 400 MHz or 500 MHz NMR spectrometer, and high-resolution mass spectra were obtained using a Thermo Scientific Q Exactive Orbitrap mass spectrometer in positive ion mode (ESI+).

Size exclusion chromatography (SEC) was performed in THF at 40 °C on a TOSOH EcoSEC HLC-8320GPC equipped with multiangle light scattering (Wyatt Technology DAWN8+) and refractive index (TOSOH) detectors. dn/dc values for block copolymers were calculated from the dn/dc value of 0.067 for PnBA and 0.085 for PMMA reported in the literature.[S4]

Scanning transmission electron microscope (STEM) images were acquired using a Ther-

mofisher FEI Talos F200C electron microscope operated at 200 kV at various magnifications. All samples were sectioned on a Leica UC6/FC6 Ultramicrotome and stained using a 2 wt.% phosphotungstic acid (PTA) and 2 wt.% benzyl alcohol solution, following a previously reported protocol,[S5] prior to imaging.

Small angle X-ray scattering (SAXS) experiments were performed on a laboratory beamline (Xeuss 2.0 HR®, Xenocs, France) using a GeniX3D microfocus sealed tube (copper) beam source with X-ray wavelength of 1.54 Å and power settings of 50 kV and 0.6 mA. A Dectris Pilatus3 R200K® detector was used with a sample to detector distance of approximately 2.5 m. The scattering experiments were collected at room temperature using silicon scatterless slits for collimation (0.5 and 0.25 mm) and integrated using XSACT software (Xenocs) to convert 2D images into one-dimensional scattering data of scattering intensity I(q) (in arbitrary units) versus q ($Å^{-1}$). Each image was produced by collecting and summing three vertical images (600 seconds each). The line eraser option was deployed to remove horizontal lines in the images due to stitching images together to obtain a wider q-range. The raw data was produced using XSACT.

Dog-bone specimens were characterized using a custom-built mechanical and optical testing setup.[S6,S7] The specimen was deformed uniaxially at a strain rate of 0.085 mm/s using two opposing translation stages. The mechanical load was recorded using a 50 lb capacity load cell (Honeywell Model 31) attached to one of the stages. A white LED pad (A4-DWT, Tikteck) was placed behind the sample and used as a light source for absorption measurements. The transmitted light was filtered using a bandpass filter with a transmission range of 550-630 nm (89-811, Edmund Optics) and recorded on a monochromatic camera (DCC1240M, Thorlabs). Both load-displacement data and optical videos were obtained during deformation. The load-displacement data were converted to engineering stress and strain. Optical data were processed by selecting a region of interest from the obtained video and calculating its average intensity in each frame. This intensity was converted to absorbance ($A = \log_{10} \frac{I_0}{I}$, where I_0 is the reference intensity of the background illumination), and the concentration

c_{act} of activated mechanophore was calculated using Beer's law,

$$A = \epsilon c_{act} l(t) \tag{1}$$

where $l(t)$ is the instantaneous sample thickness and ϵ is the previously-measured extinction coefficient of the activated mechanophore (2.0×10^4 M^{-1} cm^{-1}, ref. S3). c_{act} was finally divided by the total concentration of spiropyran in the sample to yield the percent activation of the mechanophore.[S3] The primary uncertainty in this analysis arises from uncertainty in the value of the extinction coefficient, ϵ, which was measured for UV-activated, rather than strain-activated, samples. Slight differences in the absorption spectra of the UV- and strain-activated states of the mechanphore aside,[S8] measuring UV-activated samples may lead to an under-estimate of the extinction coefficient (and, in turn, an over-estimate of the absolute activation in the strain-activated samples) if not all mechanophores were activated by the UV light prior to the extinction coefficient measurement. The good agreement of the measured extinction coefficient with other values reported in the literature,[S9,S10] however, suggests that the magnitude of the error is small. Additionally, because the same extinction coefficient was used to analyze all samples, any uncertainty in the extinction coefficient affects all samples equally and does not affect the relative trends in activation across samples.

1.2 Computational Details

The simulation method used in this study was adapted from our previously published work.[S11] In brief, the ABA triblock copolymers were modeled using a bead-spring model with parameters chosen to match the MBM system.[S11] Bidisperse blends were prepared by generating a mixture of short and long chains with $N_A = 18$ and $N_B = 30$ for the short chains and $N_A = 42$ and $N_B = 70$ for the long chains. To generate samples with equal number fractions of short and long chains, the number of chains of each type was matched, while for samples with equal weight fractions of short and long chains, the number of beads

contained in each type of chain was matched. An applied field was used to drive ordering into the expected cylindrical morphology, after which the system was equilibrated under NVT and NPT conditions. Double-well mechanophores were added in the middles of all chains and the systems were then deformed perpendicular to their characteristic domain pattern to determine their mechanical and activation behavior. The details of the simulation model, sample generation, and sample deformation are provided below.

1.2.1 Molecular dynamics model

In the molecular dynamics simulation study, all simulations were carried out in HOOMD-blue 2.9.2[S12,S13] using CUDA 10.1 on a single graphics processing unit (GPU) (NVIDIA GeForce GTX 1080Ti). The double well potential was implemented in a custom plugin[S14] for HOOMD-blue. The symmetric PMMA-*b*-PnBA-SP-PnBA-*b*-PMMA triblock copolymers were parameterized in terms of polymer glass transition temperature, melt density, Kuhn segments, and molecular weight of monomer using a coarse-grained bead-spring model. The spiropyran mechanophore unit was represented using a double-well potential with the first minimum corresponding to the deactivated state, i.e. the closed-ring spiropyran, and the second minimum representing the activated state, i.e. the open-ring merocyanine. Details of the simulation model can be found in our previous study.[S11]

1.2.2 Sample Generation

Annealed samples were obtained following a method adapted from our previously reported protocol.[S11] First, polymer chains were generated with $N_A = 18$ and $N_B = 30$ beads representing the short polymer chains and $N_A = 42$ and $N_B = 70$ beads representing the long chains. The ratio of A beads to B beads was kept constant for these two chain populations to maintain a constant volume fraction of the A block (f_A) of 0.26; this composition yields cylindrical block copolymer morphologies, which are locally comparable to the bicontinuous morphologies observed in the experimental samples. The compositions of the simulated sys-

tems are summarized in Table S8. For the samples with an equal weight fraction of short and long chains (denoted “W”), the number of beads in each population was set to be identical. Similarly, for the sample with an equal number fraction of short and long chains (denoted “N”), the number of short chains was set to be identical to the number of long chains.

Polymer chains were then randomly placed in a cuboid box of volume $V = L_x \times L_y \times L_z$ with standard periodic boundary conditions and the simulation volume density of 0.55. The system was equilibrated using dissipative particle dynamics (DPD) with the aid of an applied external field under a constant temperature (NVT) ensemble to achieve desired well-ordered microstructures.[S13,S15,S16] The periodicity of the applied external field was chosen based on the estimated domain spacing measured from direct unbiased simulations. After obtaining the targeted structures, the soft potentials used in DPD were replaced with the Lennard-Jones (LJ) and finite extensible nonlinear elastic (FENE) potentials. The additional external field was then removed and the system was cooled to a temperature between the glass transition temperatures of the two polymer components under the NPT ensemble with the Martyna-Tobias-Klein (MTK) barostat-thermostat.[S17] After sample equilibration, the spiropyran mechanophore units were incorporated into the center of the B block. Details of the protocol for sample generation and equilibration can be found in the previous study.[S11]

1.2.3 Deformation

The tensile responses of the equilibrated samples were simulated by deforming the equilibrated configurations at a constant strain rate to a maximum elongation of 400%. Deformations were carried out under an NVT ensemble using a Langevin thermostat as previously reported.[S18] All uniaxial deformations were repeated four times with independently equilibrated samples.

2 Results

2.1 Experimental Results

2.1.1 Parent Polymers

The polymers synthesized using the above methods are summarized in Table S3. The polymers are coded as PnBA-X for the PnBA midblocks and MBM-X for the MBM triblocks, where the X represents the relative polymer length (e.g., s stands for short and l for long) and whether or not the chain contains spiropyran (where lower-case letters indicate mechanically-inactive chains and upper-case letters indicate mechanically-active chains). The molecular weights of the short and long polymers, as measured by SEC (Figs. S2 and S4), differed by approximately a factor of 3. The PnBA midblocks were all found to have dispersities below 1.10, and the triblocks had dispersities around 1.20. NMR (Fig. S3) confirmed that all four triblocks had PMMA fractions of approximately 0.45. These results indicate that the parent polymers have similar compositions and well-defined, narrow molecular weight distributions, and differ primarily in the length of the rubbery PnBA midblocks.

Table S3: Parent MBM triblock copolymers synthesized in this work

**Polymer**	$\mathbf{M_{n,theo}}$ (kg/mol)[a]	$\mathbf{M_{n,NMR}}$ (kg/mol)[b]	$\mathbf{M_{n,SEC}}$ (kg/mol)	**Đ**	$\mathbf{f}_{\mathrm{PMMA}}$[c]	$\boldsymbol{\chi}\mathbf{N}$[d]
PnBA-s	31	-	27	1.06	-	-
PnBA-S	33	-	27	1.05	-	-
PnBA-l	63	-	76	1.09	-	-
PnBA-L	65	-	78	1.09	-	-
MBM-s	61	53	63	1.19	0.47	22
MBM-S	56	49	50	1.12	0.42	20
MBM-l	119	133	149	1.21	0.43	55
MBM-L	123	146	150	1.21	0.45	61

[a] Calculated from % conversions reported in Tables S1 and S2
[b] Calculated from M_n of the PnBA midblock and PnBA and PMMA mole fractions measured by NMR
[c] Calculated from mole fractions obtained from NMR, using densities of 1.18 g/cm^3 and 1.06 g/cm^3 for PMMA and PnBA, respectively[S4]
[d] χ = 0.047 at room temperature[S5]

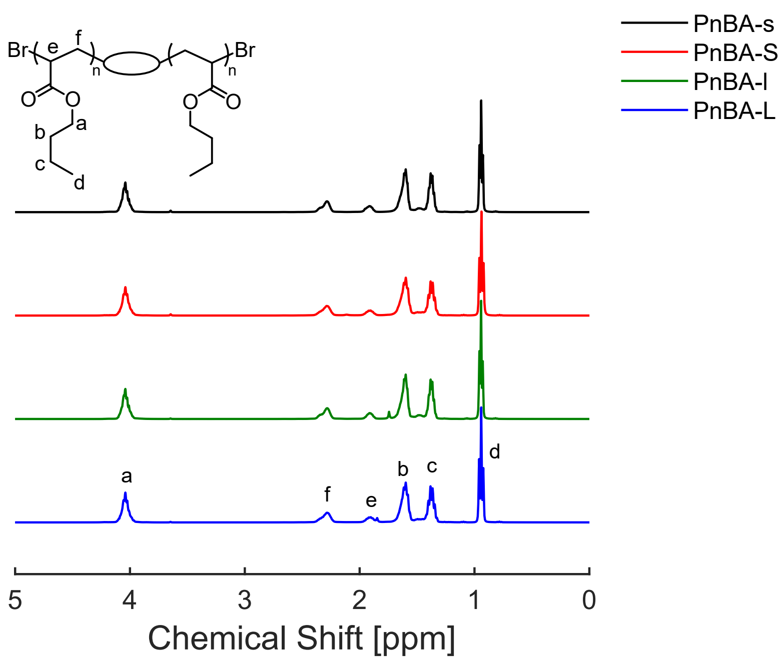

Figure S1: ^{1}H-NMR spectra of PnBA and PnBA-SP macroinitiators in $CDCl_3$ (400 MHz). Spectra are normalized to the 2H of the PnBA sidechain peak near 4.0 ppm.

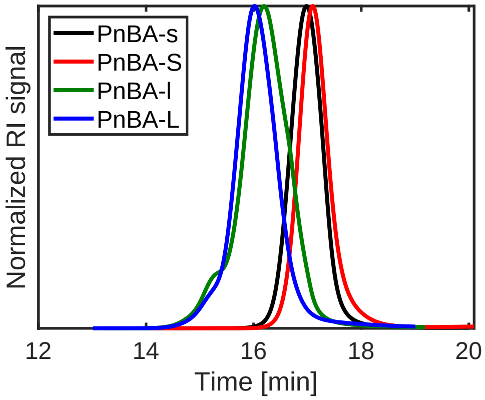

Figure S2: SEC traces of PnBA and PnBA-SP macroinitiators before chain extension, measured in THF at a flow rate of 1.0 mL/min at 40 °C.

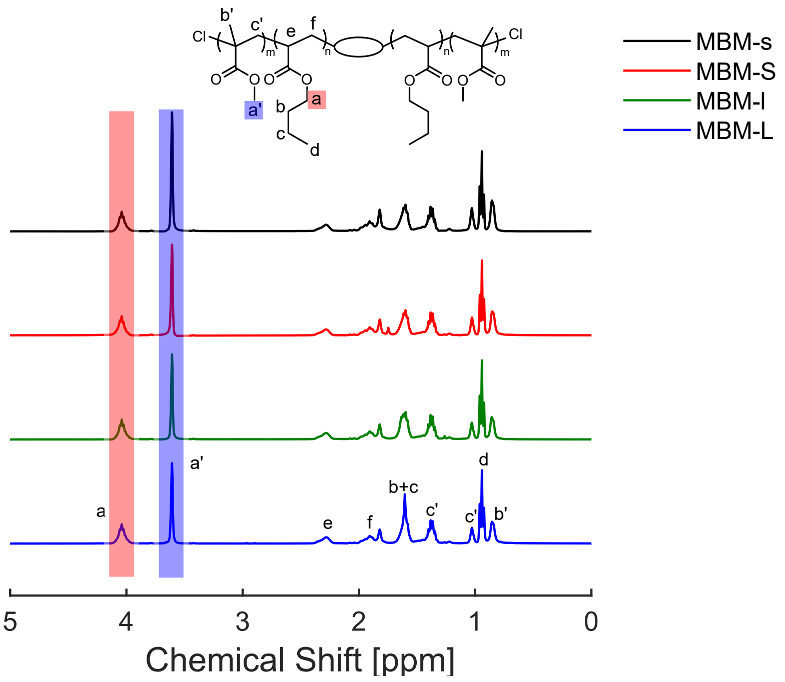

Figure S3: ^{1}H-NMR spectra of MBM and MBM-SP triblock copolymers in $CDCl_3$ (400 MHz). Spectra are normalized to the 2H of the PnBA sidechain peak (a) near 4.0 ppm. Highlighted regions indicate the peaks used to determine the volume fraction of PMMA in the triblock copolymers.

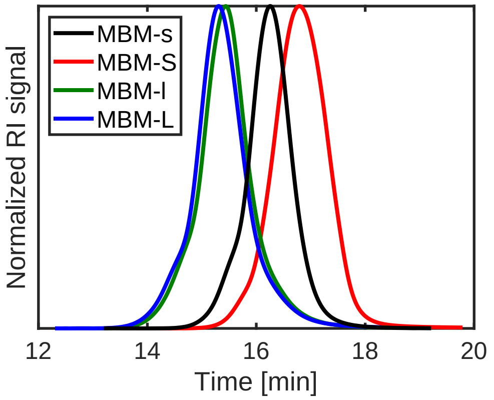

Figure S4: SEC traces of MBM and MBM-SP polymers after chain extension, measured in THF at a flow rate of 1.0 mL/min at 40 °C.

Following solvent-vapor annealing, the morphologies of the parent polymers (MBM-s, MBM-S, MBM-l, and MBM-L) were characterized via TEM and SAXS. As shown in Fig. S5, both short polymers (MBM-s and MBM-S) self-assembled into spherical morphologies with principal domain spacings of 25 and 20 nm, while the long polymers (MBM-l and MBM-L) self-assembled into cylindrical morphologies with domain spacings of 41 and 38 nm.

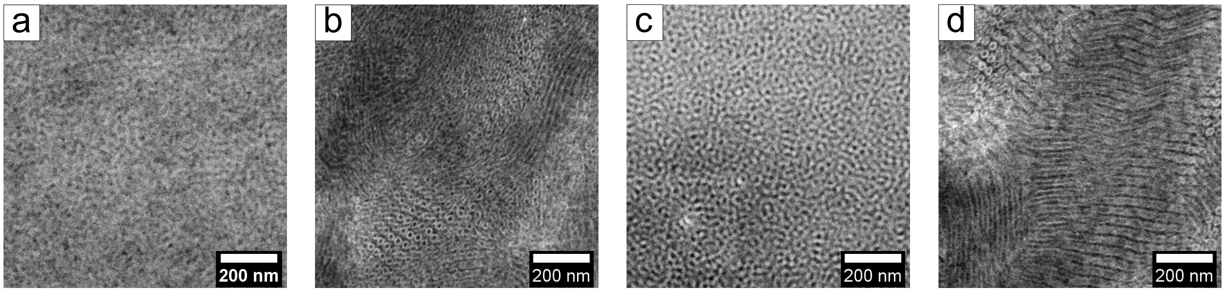

Figure S5: Scanning transmission electron microscopy (STEM) images of (a) MBM-s, (b) MBM-l, (c) MBM-S, and (d) MBM-L stained with benzyl alcohol and phosphotungstic acid. PMMA microdomains are shown in white and PnBA is shown in black.

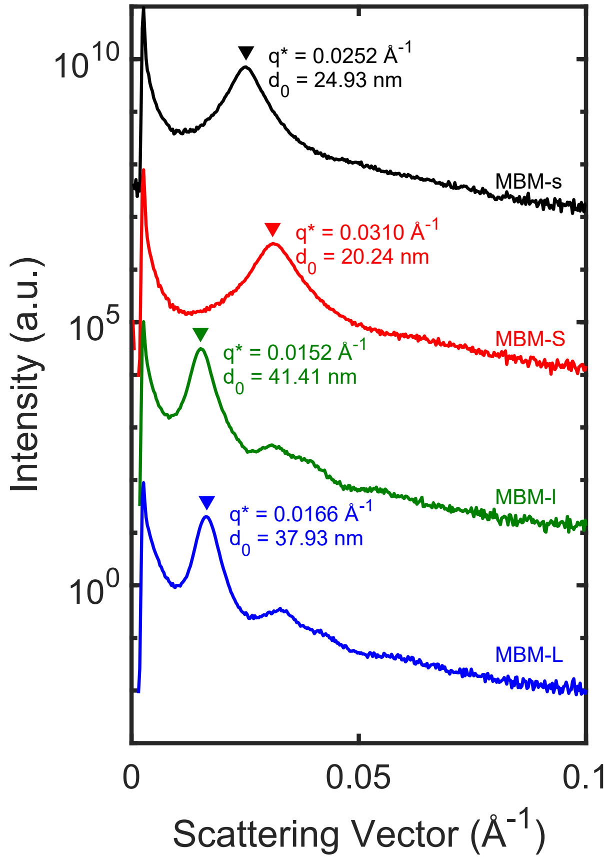

Figure S6: SAXS patterns of self-assembled MBM-s, MBM-S, MBM-l, and MBM-L parent polymers. The principal domain spacing, d_0, was calculated from the wavevector of the primary scattering peak, q^*, using $d_0 = \frac{2\pi}{q^*}$.

Stress-strain and activation-strain profiles for these polymers are shown in Fig. S7. As seen in this figure, the tensile responses of mechanically active and mechanically inactive polymers of each length were comparable. The measured stress-strain and activation-strain profiles were reproducible across multiple replicates of the same sample (Figs. S8 and S9).

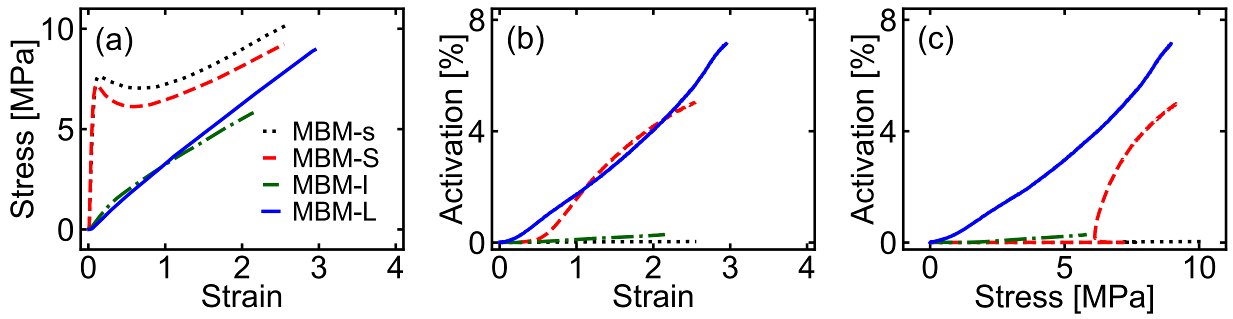

Figure S7: (a) Stress-strain, (b) activation-strain, and (c) activation-stress curves for the parent polymers.

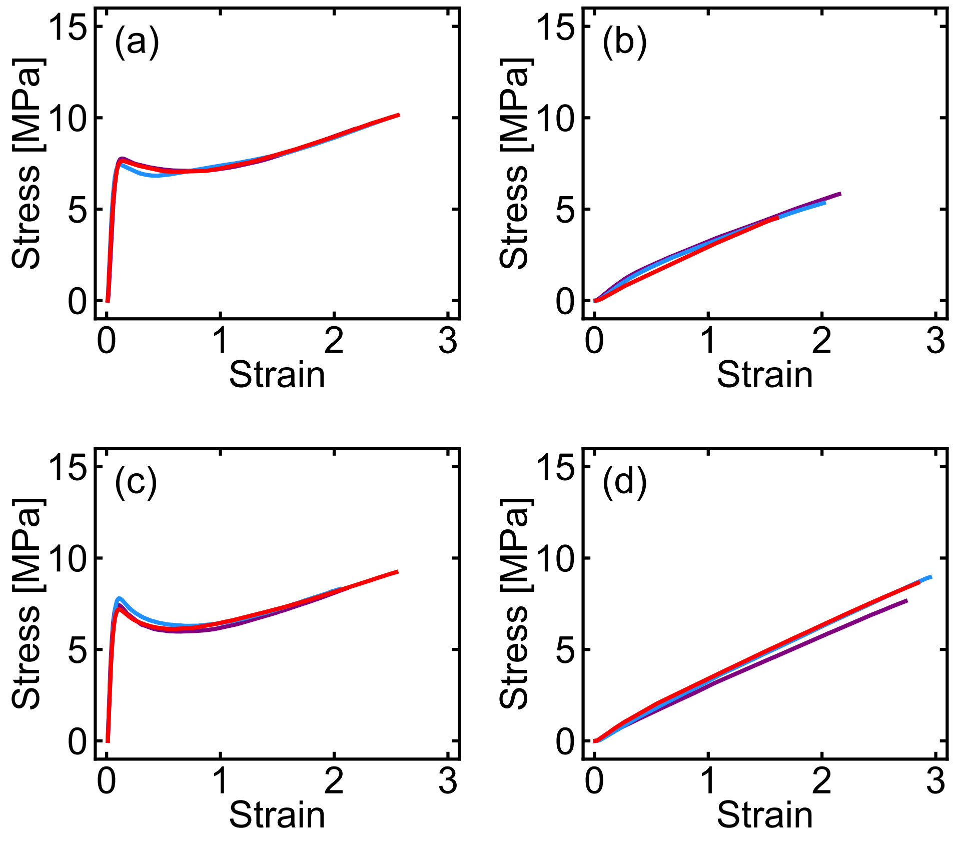

Figure S8: Stress-strain profiles of (a) MBM-s, (b) MBM-l, (c) MBM-S, (d) MBM-L obtained from measurements on different dog-bone samples.

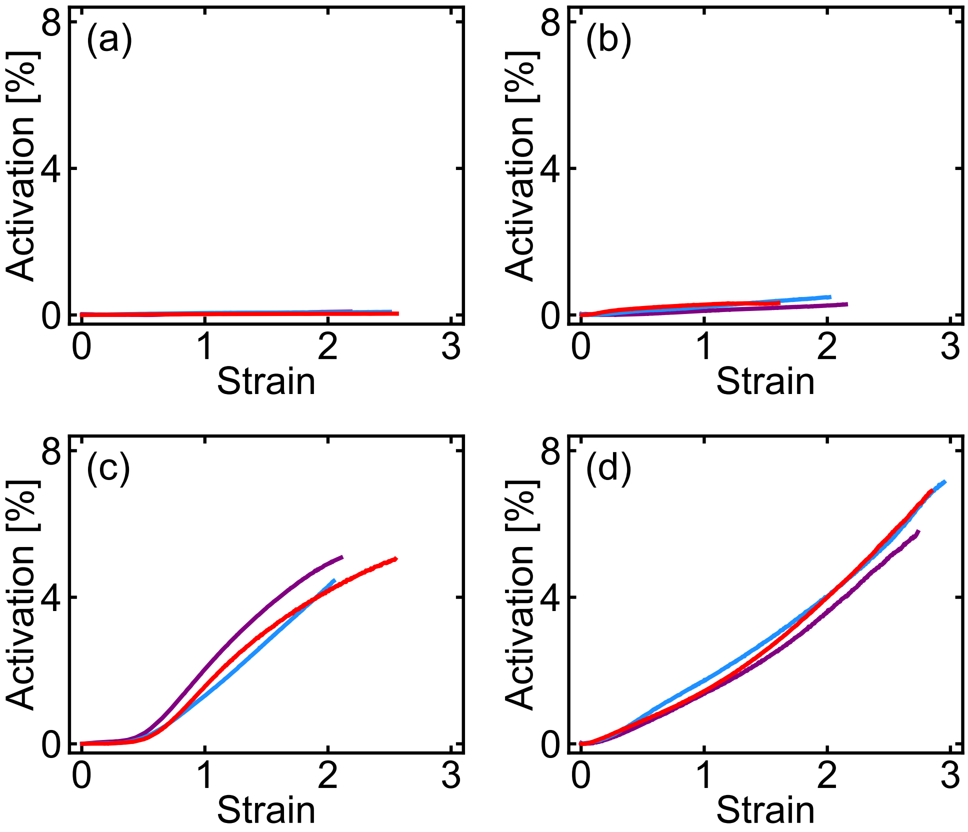

Figure S9: Activation-strain profiles of (a) MBM-s, (b) MBM-l, (c) MBM-S, (d) MBM-L, obtained from measurements on different dog-bone samples.

2.1.2 Equal Weight-Fraction Blends

The molecular weights, dispersities, and compositions of the blends prepared with equal weight fractions of short and long parent polymers are summarized in Table S4. Polymers are labeled with a "W" to indicate that the blends contain equal weight fractions of the short and long polymers, and the capitalization of the "s" and "l" labels indicate whether the short and/or long chains contain mechanophores as described above. All of the blended samples had volume fractions of PMMA of approximately 0.44. SEC traces (Fig. S10) revealed that the molecular weight distributions of all three samples were indeed bimodal, as expected, with dispersities significantly higher than those of the parent polymers.

Table S4: Molecular characteristics of blends prepared with equal weight fractions of short and long chains

**Blend ID**	$\mathbf{M}_n$ (kg/mol)[a]	$\mathbf{M}_{n,SEC}$ (kg/mol)[b]	**Đ**	$\mathbf{f}_{\mathrm{PMMA}}$[a]	**Morphology**	$\mathbf{d}_0$ (nm)[c]
W-Sl	75	74	1.60	0.43	Bicontinuous	31.1
W-sL	89	95	1.39	0.45	Bicontinuous	32.9
W-SL	75	74	1.62	0.44	Bicontinuous	30.7

[a] Calculated using the $\mathbf{M}_{n,SEC}$ and $\mathbf{f}_{\mathrm{PMMA}}$ in Table S3.
[b] Measured using SEC, with dn/dc calculated from the $\mathbf{f}_{\mathrm{PMMA}}$ values in Table S3.
[c] Calculated from SAXS (see Fig. S12).

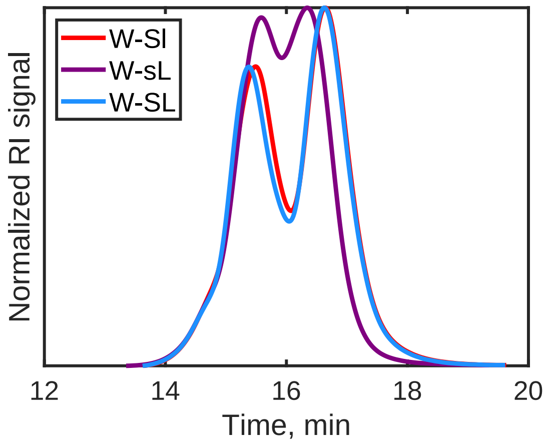

Figure S10: SEC traces of polymer blends prepared via equal weight fraction approach, measured in THF at a flow rate of 1.0 mL/min at 40 °C.

The morphologies of all three blends were characterized by STEM and SAXS. STEM im-

ages (Fig. S11) revealed that the blends all self-assembled into disordered, phase-segregated morphologies, as described in the main text. SAXS patterns (Fig. S12) indicated that the principal domain spacing was comparable in all three blends, with d_0 values of 31, 33, and 31 nm for the W-Sl, W-sL, and W-SL blends, respectively.

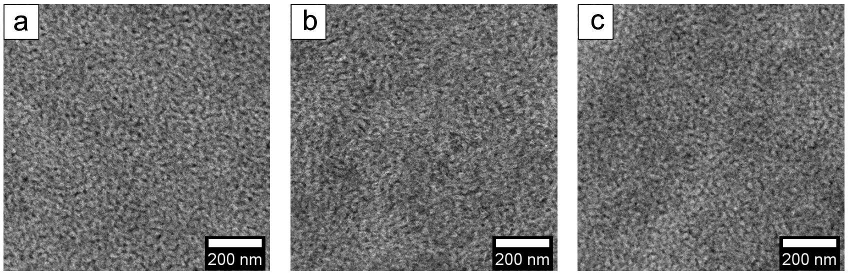

Figure S11: Scanning transmission electron microscopy (STEM) images of (a) W-Sl, (b) W-sL, and (c) W-SL stained with benzyl alcohol and phosphotungstic acid. PMMA microdomains are shown in white and PnBA is shown in black.

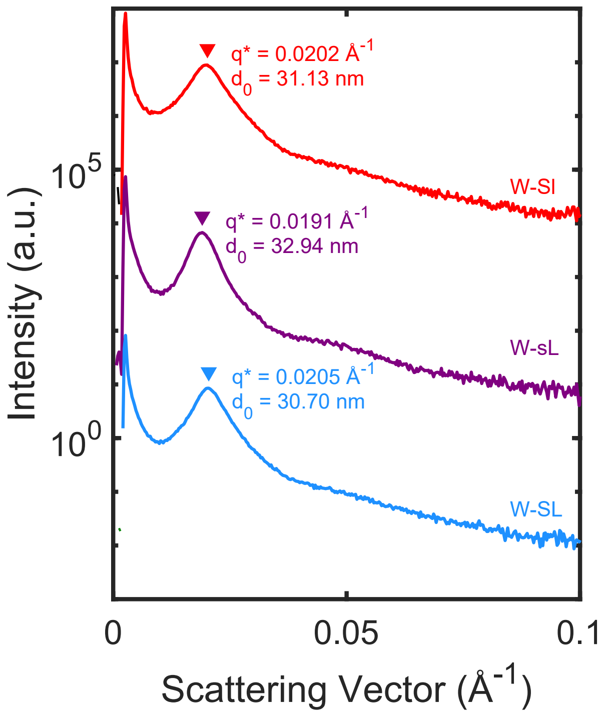

Figure S12: SAXS patterns of self-assembled W-Sl, W-sL, and W-SL blends. The principal domain spacing, d_0, was calculated from the wavevector of the primary scattering peak, q^*, using $d_0 = \frac{2\pi}{q^*}$.

Stress-activation-strain curves for all three blends are shown in Figs. S13 (where activa-

tion is normalized to the total number of chains in the sample) and S14 (where activation is normalized to the number of mechanophores in the sample). The key features of these curves are summarized in Table S5. As seen in Fig. S13a, all three samples exhibited comparable ductile behavior, with a yield point observed at $\epsilon \sim 0.1$, and their Young's Moduli were similar but increased slightly as the volume fraction of PMMA increased. As seen in Figs. S13b-c, both W-Sl and W-SL exhibited almost identical mechanochemical responses, while the W-sL samples exhibited significantly delayed activation. In these plots, the activation percentage is normalized to the total number of *chains*, such that the sum of the activation curves for W-Sl and W-sL is approximately that of W-SL. Instead normalizing the activation percentage by the number of *mechanophores* (Figs. S14) revealed that the overall activation efficiency is actually higher in the W-Sl sample than in the W-SL blend. As discussed in the main text, these results indicate that the activation is dominated primarily by the short elastic strands, and that the highest activation efficiencies are achieved when the mechanophores are placed only in the short strands. These results were reproducible across multiple tensile samples for each polymer blend (Figs. S15 and S16).

Table S5: Mechanical and activation properties of blends prepared with equal weight fractions of short and long chains

**Blend ID**	**Young's Modulus** (MPa)	**Strain onset**	**Stress onset** (MPa)
W-Sl	87±4[a]	0.67±0.08	6.1±0.6
W-sL	132±9	2.25±0.02	8.9±0.3
W-SL	109±3	0.68±0.03	6.3±0.3

[a] Uncertainty was determined from the standard deviation of 3 or 4 different measurements.

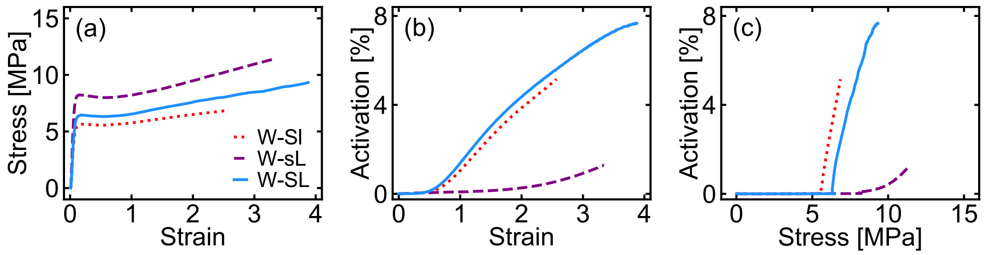

Figure S13: (a) Stress-strain (b) activation-strain, and (c) activation-stress curves for MBM polymer blends prepared with equal weight fractions of short and long polymers. The percent activation is normalized to the total number of polymer chains in the sample. All stress/strain values are engineering stress and engineering strain unless otherwise noted.

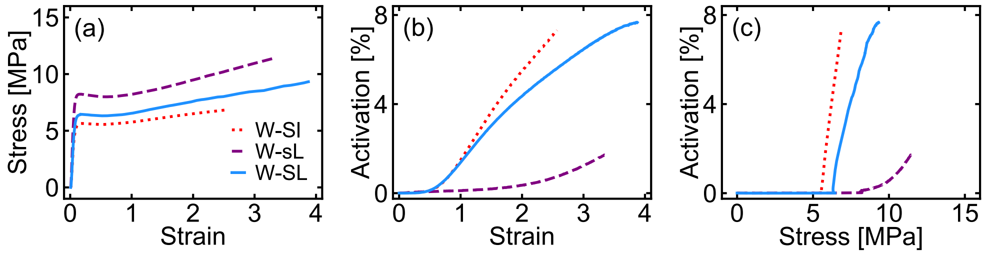

Figure S14: (a) Stress-strain (b) activation-strain, and (c) activation-stress curves for MBM polymer blends prepared with equal weight fractions of short and long polymers. The percent activation is normalized to the number of mechanically active polymer chains in the sample.

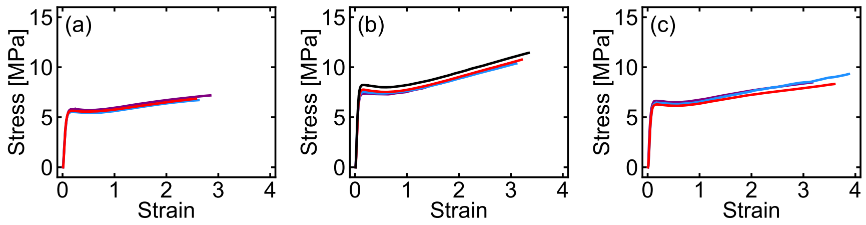

Figure S15: Stress-strain profiles of (a) W-Sl (b) W-sL (c) W-SL obtained from measurements on different dog-bone samples.

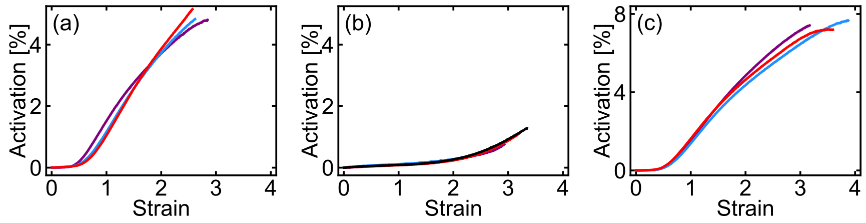

Figure S16: Activation-true strain profiles of (a) W-Sl (b) W-sL (c) W-SL obtained from measurements on different dog-bone samples.

2.1.3 Equal Number-Fraction Blends

All data in the main text is reported for samples with equal weight fractions of short and long chains. Analogous experiments were also conducted on samples with equal number fractions of short and long chains, denoted N-Sl, N-sL, and N-SL. The compositions, molecular weight distributions, and morphologies of the resulting samples are shown in Table S6 and Figs. S17-S19. The corresponding tensile data is shown in Figs. S20-S23. As seen in this data, the trends observed in the samples with equal number fractions of short and long chains were essentially the same as those reported for samples with equal weight fractions of short and long chains as reported in the main text.

Table S6: Molecular characteristics of blends prepared via equal number fraction method

**Blend ID**	$\mathbf{M}_n$ (kg/mol)[a]	$\mathbf{M}_{n,SEC}$ (kg/mol)[b]	**Đ**	$\mathbf{f}_{\text{PMMA}}$[a]	**Morphology**	$\mathbf{d}_0$ (nm)[c]
N-Sl	100	95	1.50	0.44	Bicontinuous	37.9
N-sL	106	106	1.37	0.44	Bicontinuous	38.6
N-SL	100	98	1.48	0.45	Bicontinuous	39.3

[a] Calculated using the $\mathbf{M}_{n,SEC}$ and $\mathbf{f}_{\text{PMMA}}$ in Table S3.
[b] Measured using SEC, with dn/dc calculated from the $\mathbf{f}_{\text{PMMA}}$ values in Table S3.
[c] Calculated from SAXS (see Fig. S19).

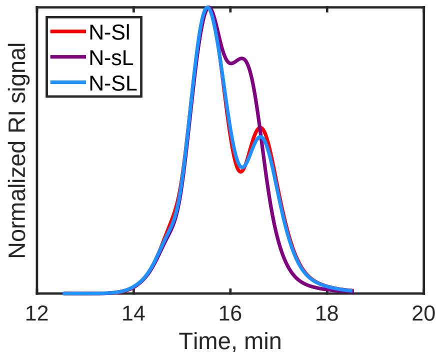

Figure S17: SEC traces of polymer blends prepared via equal number fraction approach, measured in THF at a flow rate of 1.0 mL/min at 40 °C.

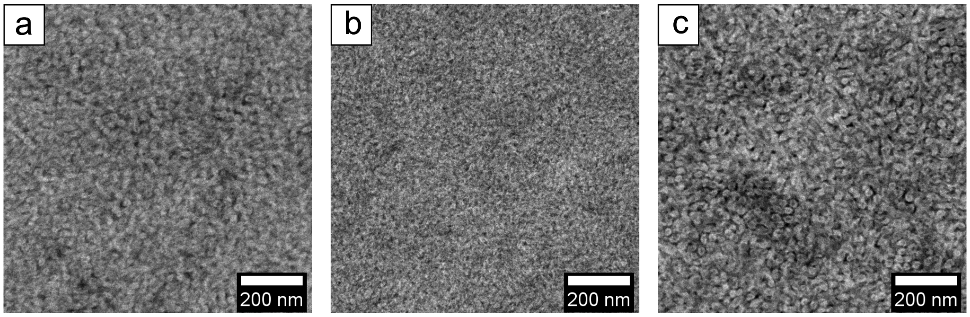

Figure S18: Scanning transmission electron microscopy (STEM) images of the polymer blends with equal number fractions stained with benzyl alcohol and phosphotungstic acid. PMMA microdomains are shown in white and PnBA is shown in black. (a) N-Sl (b) N-sL (c) N-SL.

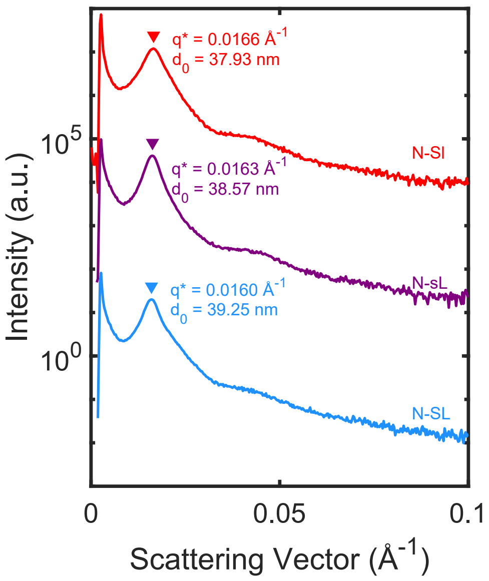

Figure S19: SAXS patterns of self-assembled N-Sl, N-sL, and N-SL blends. The principal domain spacing, d_0, was calculated from the wavevector of the primary scattering peak, q^*, using $d_0 = \frac{2\pi}{q^*}$.

Table S7: Mechanical and activation properties of blends prepared via equal number fraction method

**Blend ID**	**Young's Modulus** (MPa)	**Strain onset**	**Stress onset** (MPa)
N-Sl	86±9[a]	0.70±0.03	5.9±0.4
N-sL	109±2	1.75±0.05	8.5±0.6
N-SL	85±4	0.70±0.03	5.6±0.2

[a] The uncertainty was determined from the standard deviation of 3 or 4 different dog-bone measurements.

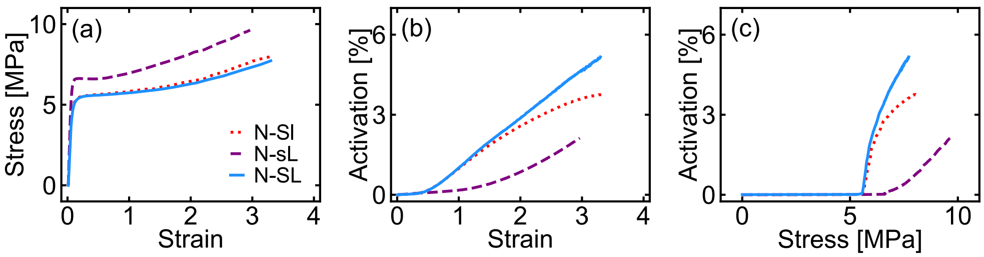

Figure S20: (a) Stress-strain (b) activation-strain, and (c) activation-stress curves for all MBM polymer blends prepared with equal number fractions of short and long chains. The percent activation is normalized to the total number of polymer chains in the sample.

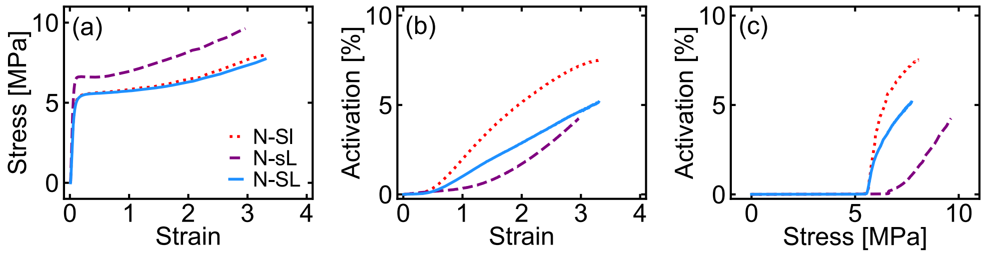

Figure S21: (a) Stress-strain (b) activation-strain, and (c) activation-stress curves for all MBM polymer blends prepared with equal number fractions of short and long chains. The percent activation is normalized to the number of mechanically active polymer chains in the sample.

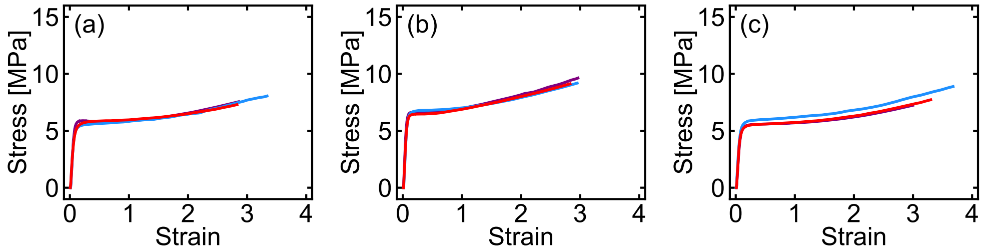

Figure S22: Stress-strain profiles of (a) N-Sl (b) N-sL (c) N-SL obtained from measurements on different dog-bone samples.

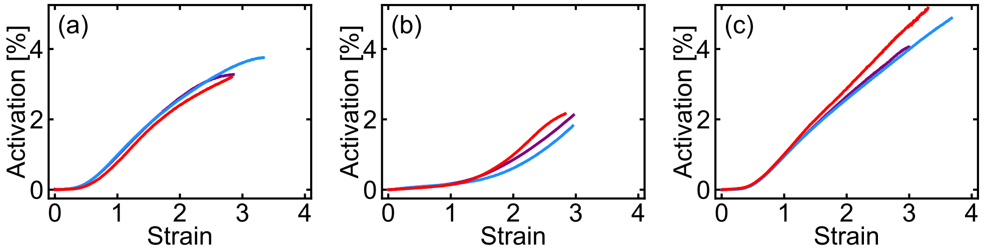

Figure S23: Activation-strain profiles of (a) N-Sl (b) N-sL (c) N-SL obtained from measurements on different dog-bone samples.

2.2 Computational Results

2.2.1 Equilibrated Morphologies

As in the experiments described above, MD simulations were also carried out on blends with either equal weight fractions or equal number fractions of short and long chains. Snapshots of representative equilibrated morphologies for both types of blends are shown in Figure S24, and their key characteristics are summarized in Table S8. The overall tie fraction was found to be 0.58 ± 0.01 in the system with equal weight fractions of short and long chains, and 0.51 ± 0.01 in the system with equal number fractions of short and long chains, in good agreement with the value predicted by SCFT.[S19] The tie fractions were different for the short and long chains within each sample, however, with the short chains having tie fractions somewhat lower than the average and the long chains having tie fractions somewhat higher than the average. As in the experiments, the domain spacing was larger in the blends with equal number fractions of short and long chains.

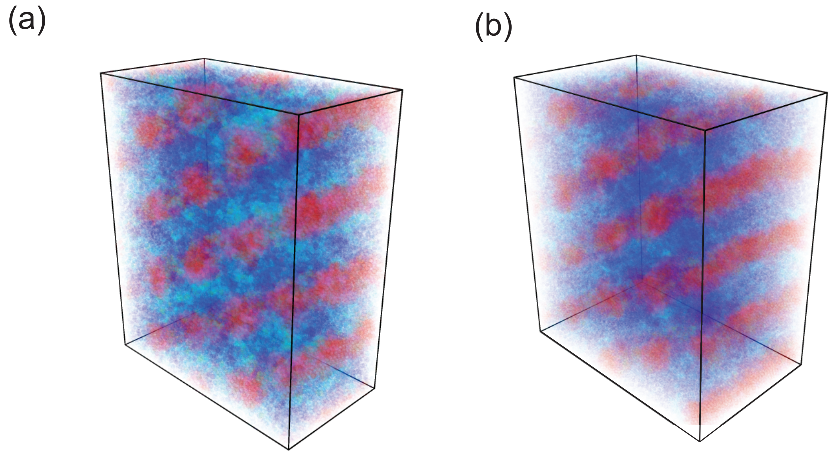

Figure S24: Representative snapshots of equilibrated morphologies obtained for (a) blends with equal weight fractions of short (cyan) and long (blue) chains, and (b) blends with equal number fractions of short and long chains.

Table S8: Polymer samples, blending method, box sizes, number of chains.

System	Blend	Morphology	V, σ^3	Tie fraction	Spacing, σ	N_{Long}	N_{Short}
$A_9B_{30}A_9$+$A_{21}B_{70}A_{21}$	W	cylindrical	$67 \times 58 \times 33$	0.58 ± 0.01 [a]<br>0.76 ± 0.01 (Long)<br>0.50 ± 0.01 (Short)	15	413	965
$A_9B_{30}A_9$+$A_{21}B_{70}A_{21}$	N	cylindrical	$84 \times 73 \times 44$	0.51 ± 0.01[a]<br>0.68 ± 0.01 (Long)<br>0.36 ± 0.01 (Short)	18	1219	1219

[a] The uncertainty was determined from the standard deviation from four independently equilibrated samples.

2.2.2 Tensile and Activation Responses

Equal Weight-Fraction Blends Stress-strain, activation-strain, and activation-stress curves from simulations of samples with equal weight fractions of short and long chains are shown in Fig. S25. As seen in Fig. S25a, the total stress carried by the short and the long chains is almost identical at low true strain but is dominated by the long chains at high true strain. On a per-chain basis, however, since the number of long chains is lower, the long chains bear more stress than the short chains. The activation-strain plot in Fig. S25b indicates that the overall activation closely follows that of the short chains, and activation of the long chains only turning on at high strain. The maximum activation obtained in this bidisperse system is ∼30%, which is comparable to that obtained in the monodisperse cylindrical system we reported earlier with an elastic strand length of $N_B = 50$.[S11] Finally, as shown in Fig. S25c, activation of the short chains turns on at lower stress than the activation of the long chains.

As noted in the main text, the MD simulations offer the opportunity to investigate the activation of different chain types (loop, tie, and hook) within the sample, and to analyze how activation is related to other molecular-scale features such as chain conformation and alignment. As seen in Fig. S26, the short tie chains are more likely to turn into dangling chains as the sample is extended than the long tie chains are. The activation of the short chains is also higher than the activation of the long chains across different chain types (Fig. S27). With respect to chain conformation, we find that the average end-to-end distance

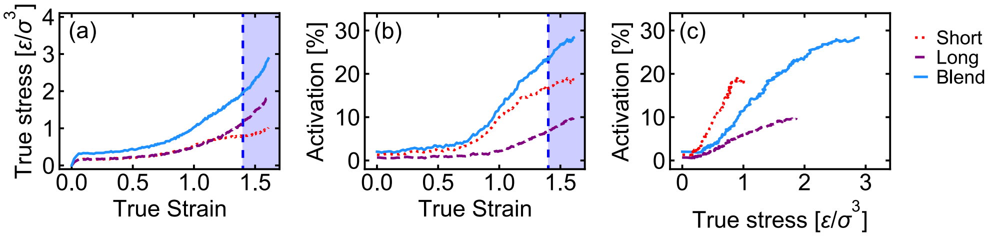

Figure S25: (a) True stress-true strain (b) activation-true strain and (c) activation-true stress curves from MD simulations of blends with equal weight fractions of short and long chains and their subdivision based on the chain length. Each curve represents an average from four independent simulation runs. The percent activation is normalized to the number of polymer chains in the sample. The shaded areas indicate strains at which cavitation was observed.

R_e of the midblocks increased as the strain increased (Fig. S28a). The initial extension of the short tie chains was higher than the initial extension of the long tie chains, and the short tie chains reached their maximum extension earlier. Finally, with respect to chain alignment, we find that the activated tie chains are more aligned to the pulling direction than the un-activated tie chains (Fig. S28b), and the activated short tie chains show better alignment than the activated long tie chains.

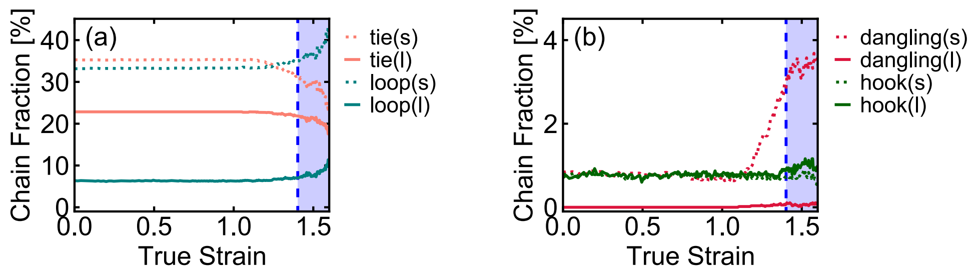

Figure S26: Fractions of (a) tie and loop chains and (b) dangling and hooked chains over the course of MD simulations on blends with equal weight fractions of short and long chains. The shaded areas indicate strains at which cavitation was observed.

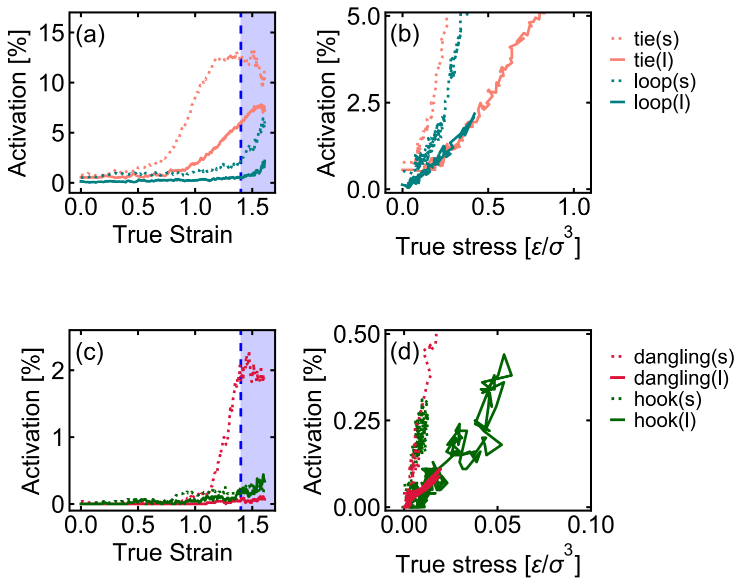

Figure S27: (a,c) Activation-strain and (b,d) activation-stress curves for (a,b) tie and loop chains and (c,d) hooked and dangling chains obtained from simulations of blends with equal weight fractions of short and long chains. Each curve represents an average from four independent simulation runs. The shaded areas indicate strains at which cavitation was observed.

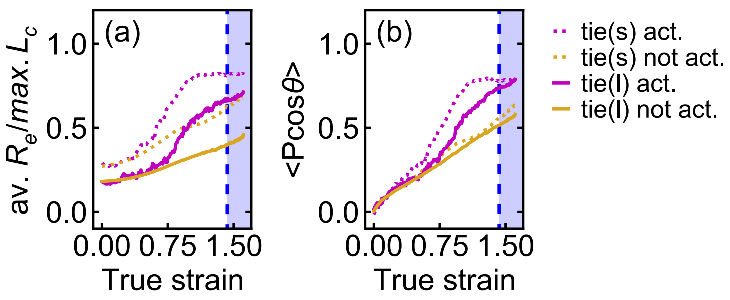

Figure S28: (a) Average end-to-end distance R_e of the midblocks normalized to their contour length L_c and (b) BB bond orientation parameter $\langle P\cos\theta\rangle$ for tie chains obtained from simulations of blends with equal weight fractions of short and long chains. Note that $\langle P\cos\theta\rangle \equiv 1$ indicates that the bonds are perfectly aligned with the pulling direction.[S20] The shaded areas indicate strains at which cavitation was observed.

Equal Number-Fraction Blends Analogous simulations were also conducted on the systems with equal number fractions of short and long chains. The activation-stress-strain profiles of these systems, and the corresponding analysese by chain type, are summarized in Figs. S29-S32. As seen in these figures, the trends observed in the samples with equal number fractions of short and long chains were essentially the same as those reported for

samples with equal weight fractions of short and long chains as reported in the main text.

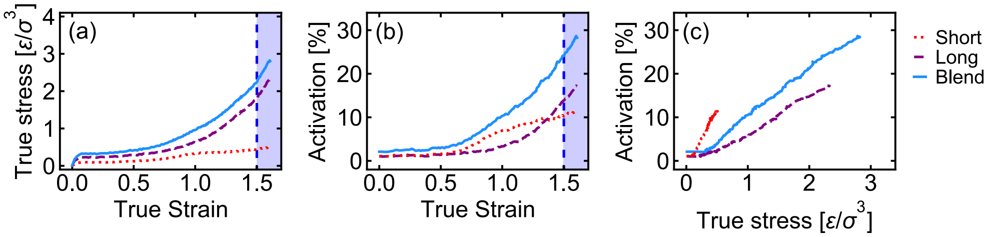

Figure S29: (a) True stress-true strain (b) activation-true strain and (c) activation-true stress curves from MD simulations of blends with equal number fractions of short and long chains and their subdivision based on the chain length. Each curve represents an average from four independent simulation runs. The percent activation is normalized to the number of polymer chains in the sample. The shaded areas indicate strains at which cavitation was observed.

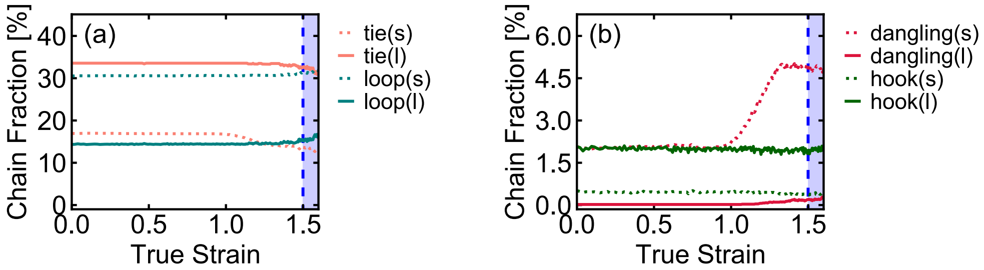

Figure S30: Fractions of (a) tie and loop chains and (b) dangling and hooked chains over the course of MD simulations on blends with equal number fractions of short and long chains. The shaded areas indicate strains at which cavitation was observed.

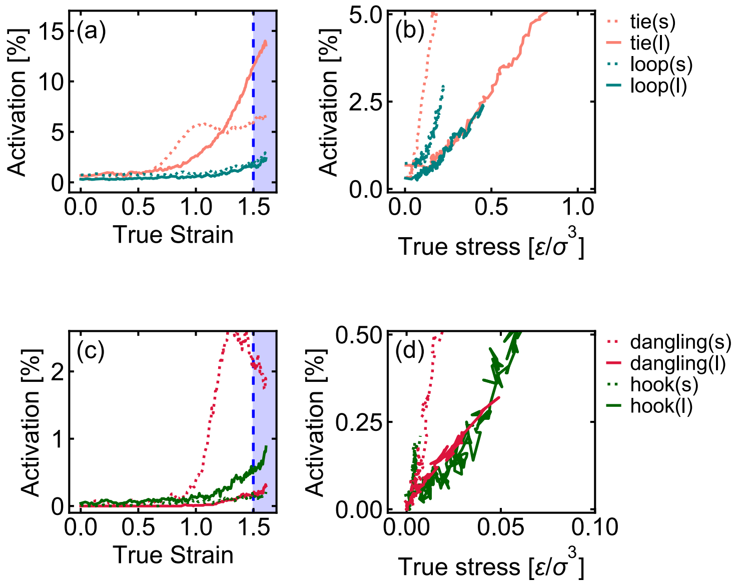

Figure S31: (a,c) Activation-strain and (b,d) activation-stress curves for (a,b) tie and loop chains and (c,d) hooked and dangling chains obtained from simulations of blends with equal number fractions of short and long chains. Each curve represents an average from four independent simulation runs. The shaded areas indicate strains at which cavitation was observed.

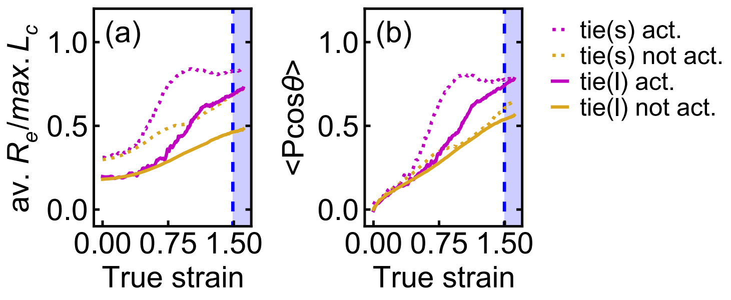

Figure S32: (a) Average end-to-end distance R_e of the midblocks normalized to their contour length L_c and (b) BB bond orientation parameter $\langle P\cos\theta\rangle$ for tie chains obtained from simulations of blends with equal number fractions of short and long chains. The shaded areas indicate strains at which cavitation was observed.

2.3 Comparison of Experimental and Computational Results

A direct comparison of the experimental and computational stress-strain and activation-strain curves for samples with equal weight fractions of short and long chains is shown in Figure S33. As seen in this figure, the simulations capture the key features of the experimental data. In both the experimental and simulation data, the onset of activation in samples

with mechanophores in either only the short chains or in both the short and long chains is observed near $\epsilon \approx 1$. While direct comparison of the stress onsets is not possible because the coarse-grained simulation units of stress (ϵ/σ^3) cannot be rigorously converted to MPa, the simulations do qualitatively predict the activation of short chains at lower stress than required for activation of the long chains. The primary differences between the simulation and experimental activation profiles are (1) the higher total activation measured in the simulations, which we attribute in large part to the perfect alignment of the cylindrical morphology used in the simulations (in contrast to the experimental morphologies, which are much more isotropic), and (2) the sharper onset of activation as a function of stress in the experiments than in the simulations, which we attribute to the fact that activation in the experimental systems occurs only after the yield point and plastic deformation of the glassy domains (which is not captured in the simulation model).

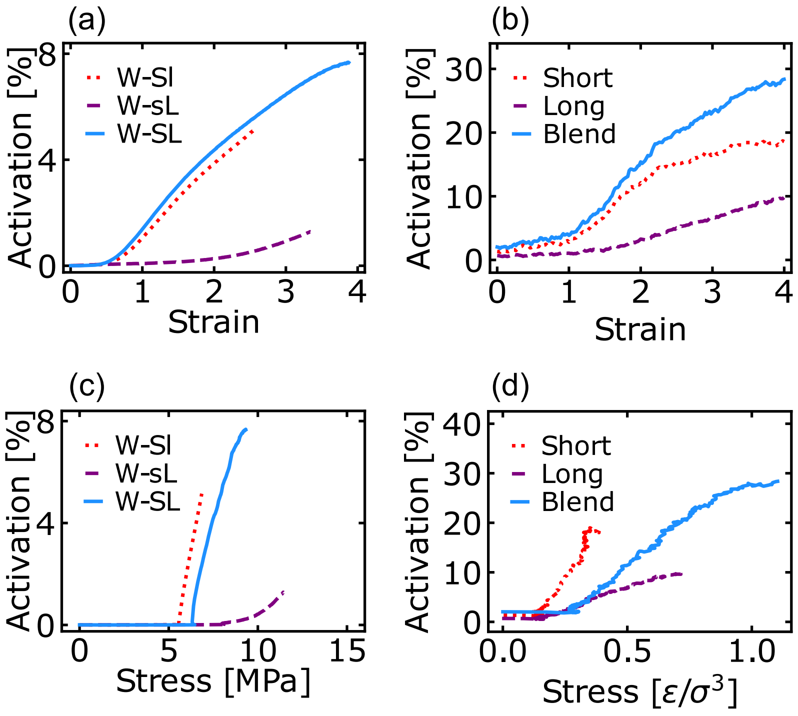

Figure S33: Comparison of (a,c) experimental and (b,d) simulated activation profiles as a function of (a,b) engineering strain and (c,d) engineering stress for samples with equal weight fractions of short and long chains.

References

(S1) Heng, S.; Mak, A. M.; Stubing, D. B.; Monro, T. M.; Abell, A. D. Dual Sensor for Cd(II) and Ca(II): Selective Nanoliter-Scale Sensing of Metal Ions. *Analytical Chemistry* **2014**, *86*, 3268–3272, DOI: 10.1021/ac500619z.

(S2) O'Bryan, G.; Wong, B. M.; McElhanon, J. R. Stress Sensing in Polycaprolactone Films via an Embedded Photochromic Compound. *ACS Applied Materials & Interfaces* **2010**, *2*, 1594–1600, DOI: 10.1021/am100050v.

(S3) Huo, Z.; Arora, S.; Kong, V. A.; Myrga, B. J.; Statt, A.; Laaser, J. E. Effect of Polymer Composition and Morphology on Mechanochemical Activation in Nanostructured Triblock Copolymers. *Macromolecules* **2023**, *56*, 1845–1854, DOI: 10.1021/acs.macromol.2c02475.

(S4) Lu, W.; Goodwin, A.; Wang, Y.; Yin, P.; Wang, W.; Zhu, J.; Wu, T.; Lu, X.; Hu, B.; Hong, K.; Kang, N.-G.; Mays, J. All-acrylic superelastomers: facile synthesis and exceptional mechanical behavior. *Polymer Chemistry* **2018**, *9*, 160–168, DOI: 10.1039/c7py01518f.

(S5) Ruzette, A.-V.; Tencé-Girault, S.; Leibler, L.; Chauvin, F.; Bertin, D.; Guerret, O.; Gérard, P. Molecular Disorder and Mesoscopic Order in Polydisperse Acrylic Block Copolymers Prepared by Controlled Radical Polymerization. *Macromolecules* **2006**, *39*, 5804–5814, DOI: 10.1021/ma060541u.

(S6) Celestine, A.-D. N.; Beiermann, B. A.; May, P. A.; Moore, J. S.; Sottos, N. R.; White, S. R. Fracture-induced activation in mechanophore-linked, rubber toughened PMMA. *Polymer* **2014**, *55*, 4164–4171, DOI: 10.1016/j.polymer.2014.06.019.

(S7) Standard Test Method for Tensile Properties of Plastics. DOI: https://www.astm.org/d0638-14.html.

(S8) Kim, T. A.; Robb, M. J.; Moore, J. S.; White, S. R.; Sottos, N. R. Mechanical Reactivity of Two Different Spiropyran Mechanophores in Polydimethylsiloxane. *Macromolecules* **2018**, *51*, 9177–9183, DOI: 10.1021/acs.macromol.8b01919.

(S9) Zhang, S.; Zhang, Q.; Ye, B.; Li, X.; Zhang, X.; Deng, Y. Photochromism of Spiropyran in Ionic Liquids: Enhanced Fluorescence and Delayed Thermal Reversion. *The Journal of Physical Chemistry B* **2009**, *113*, 6012–6019, DOI: 10.1021/jp9004218.

(S10) Krohm, F.; Kind, J.; Savka, R.; Janßen, M. A.; Herold, D.; Plenio, H.; Thiele, C. M.; Andrieu-Brunsen, A. Photochromic spiropyran- and spirooxazine-homopolymers in mesoporous thin films by surface initiated ROMP. *Journal of Materials Chemistry C* **2016**, *4*, 4067–4076, DOI: 10.1039/c5tc04054j.

(S11) Huo, Z.; Skala, S. J.; Falck, L. R.; Laaser, J. E.; Statt, A. Computational Study of Mechanochemical Activation in Nanostructured Triblock Copolymers. *ACS Polymers Au* **2022**, *2*, 467–477, DOI: 10.1021/acspolymersau.2c00031.

(S12) Anderson, J. A.; Glaser, J.; Glotzer, S. C. HOOMD-blue: A Python package for high-performance molecular dynamics and hard particle Monte Carlo simulations. *Computational Materials Science* **2020**, *173*, 109363, DOI: 10.1016/j.commatsci.2019.109363.

(S13) Phillips, C. L.; Anderson, J. A.; Glotzer, S. C. Pseudo-random number generation for Brownian Dynamics and Dissipative Particle Dynamics simulations on GPU devices. *Journal of Computational Physics* **2011**, *230*, 7191–7201, DOI: https://doi.org/10.1016/j.jcp.2011.05.021.

(S14) Plugin for HOOMD-blue: azplugins. **v0.10.0, https://github.com/mphowardlab/azplugins**, DOI: https://github.com/mphowardlab/azplugins.

(S15) Groot, R. D.; Warren, P. B. Dissipative particle dynamics: Bridging the gap between atomistic and mesoscopic simulation. *The Journal of Chemical Physics* **1997**, *107*, 4423–4435, DOI: 10.1063/1.474784.

(S16) Padmanabhan, P.; Martinez-Veracoechea, F.; Escobedo, F. A. Computation of Free Energies of Cubic Bicontinuous Phases for Blends of Diblock Copolymer and Selective Homopolymer. *Macromolecules* **2016**, *49*, 5232–5243, DOI: 10.1021/acs.macromol.6b00123.

(S17) Martyna, G. J.; Tobias, D. J.; Klein, M. L. Constant pressure molecular dynamics algorithms. *The Journal of Chemical Physics* **1994**, *101*, 4177–4189, DOI: 10.1063/1.467468.

(S18) Aoyagi, T.; Honda, T.; Doi, M. Microstructural study of mechanical properties of the ABA triblock copolymer using self-consistent field and molecular dynamics. *The Journal of Chemical Physics* **2002**, *117*, 8153–8161, DOI: 10.1063/1.1510728.

(S19) Matsen, M. W.; Thompson, R. B. Equilibrium behavior of symmetric ABA triblock copolymer melts. *The Journal of Chemical Physics* **1999**, *111*, 7139–7146, DOI: 10.1063/1.480006.

(S20) Ward, I. M., Ed. *Structure and Properties of Oriented Polymers*; Springer Netherlands, 1997; DOI: 10.1007/978-94-011-5844-2.
